# Evaluation of *Cannabis sativa* L. Callus Extract as a Novel Cosmetic Ingredient with Dual Anti-Inflammatory and Antioxidant Effects

**DOI:** 10.3390/plants14071148

**Published:** 2025-04-07

**Authors:** Ga-Ram Yu, Da-Hoon Kim, Hyuck Kim, Dong-Woo Lim

**Affiliations:** 1 Department of Diagnostics, College of Korean Medicine, Dongguk University, Goyang-si 10326, Republic of Korea; kalama2@dongguk.edu (G.-R.Y.); hk_ceo@topo.co.kr (H.K.); 2 Institute of Korean Medicine, Dongguk University, Goyang-si 10326, Republic of Korea; 3 TOPO Lab. Co., Ltd., Goyang-si 10326, Republic of Korea; kimdahoon@topo.co.kr

**Keywords:** *Cannabis sativa* L, plant callus, cosmetic ingredient, NRF2-NF-κB, callus elicitation

## Abstract

The plant callus culture technique is an emerging source of bioactive compounds with potential applications in cosmetics and pharmaceuticals. Callus-derived extracts contain high concentrations of secondary metabolites with significant antioxidant and anti-inflammatory properties when elicited. *Cannabis sativa* L. has been used for its medicinal effects; however, the potential of its *C. sativa* callus extract (CCE) for cosmetic applications remains unexplored. Callus from *C. sativa* was induced in vitro using a Murashige and Skoog (MS) medium supplemented with Thidiazuron (TDZ) and naphthalene acetic acid (NAA). The extract was analyzed for its bioactive composition using high-performance liquid chromatography (HPLC). The antioxidant activity was assessed using the DPPH radical scavenging assay. The anti-inflammatory effects were evaluated in lipopolysaccharides (LPS)-stimulated RAW264.7 macrophages by measuring nitric oxide (NO) production, DAF-2 fluorescence intensity, released cytokine levels, and protein expression of inflammatory mediators via ELISA, Western blot, and immunofluorescence assays. CCE demonstrated significant radical scavenging activity. CCE effectively suppressed LPS-induced NO production and reduced pro-inflammatory cytokine levels. Western blot analysis revealed that CCE inhibited NF-κB nuclear translocation while upregulating NRF2-mediated antioxidant responses. Furthermore, HPLC analysis confirmed the presence of cannabinoids, which could potentially be associated with the modulation of inflammatory pathways through the endocannabinoid system. This study provides evidence that CCE possesses notable antioxidant and anti-inflammatory properties, making it a promising ingredient for cosmetic formulations targeting oxidative stress and inflammatory skin conditions.

## 1. Introduction

Plants are primarily composed of meristematic (dividing) tissues and permanent (non-dividing) tissues [1]. Plant callus is an undifferentiated, amorphous mass of cells which forms around wounded areas of a plant where cell division is highly active [2]. When meristematic cells are cultured in a nutrient medium, callus formation occurs, followed by the development of adventive embryos, which can differentiate into whole plants [3,4]. Callus can be induced from various plant parts, including stems, leaves, roots, and embryos [5].

Callus culture is widely utilized in micropropagation, a technique that enables rapid plant reproduction under controlled conditions [6]. Notably, this method is particularly efficient for mass-producing valuable plant species, including rare and endangered plants [7].

Due to its highly proliferative nature, callus contains high concentrations of bioactive compounds [8], exhibiting potent anti-inflammatory [9], antioxidant [10], anti-wrinkle [11], and whitening effects [12]. In particular, various chemical elicitors can modulate the production of bioactive ingredients in plant callus, known as plant secondary metabolites [13]. In short, plant callus contains a diverse range of phytochemicals beneficial to human skin, making it a valuable source of novel cosmeceutical and bio-based cosmetic ingredients [14].

Inflammatory conditions of the skin can be triggered by external factors such as UV radiation, pollution, and microbial invasion [15], as well as internal factors including oxidative stress and immune responses [16]. Prolonged inflammation compromises the skin barrier, increasing susceptibility to various dermatological disorders such as atopic dermatitis, acne, and rosacea [17].

Notably, oxidative stress exacerbates inflammatory responses leading to skin aging and hyperpigmentation [18]. The skin employs antioxidant defense mechanisms to neutralize reactive oxygen species (ROS) [19]. Enhancing these antioxidant pathways plays a crucial role in improving skin health and preventing inflammation-related damage.

The NF-κB signaling pathway is a key regulator of inflammatory responses in the skin leading to the expression of pro-inflammatory mediators [20]. Meanwhile, the NRF2 pathway serves as a crucial antioxidant defense mechanism, promoting the transcription of genes involved in cellular detoxification such as heme oxygenase-1 (HO-1) [21]. Therefore, several studies have highlighted the importance of modulating NF-κB and NRF2 signaling pathways in treating skin conditions [21,22,23]. There is a growing demand for safer and more effective cosmetic ingredients that provide skin benefits without causing adverse effects [24].

*Cannabis sativa* L. is an annual herbaceous plant of the Cannabaceae family, naturally occurring in various regions worldwide [25]. In the past, *C. sativa* has long been used in folk medicine to treat skin diseases and conditions including skin burns [26], pruritus, and eczema [27]. The global legalization trend of *C. sativa* has garnered significant attention, promoting its development for various industrial applications, including medicine and cosmetics [28,29]. *C. sativa* contains cannabinoids, which are known to modulate inflammatory signals through unique endocannabinoid pathways. As a result, there is growing interest in its potential as a cosmetic ingredient [30]. Various parts of *C. sativa* and different extraction methods have been explored for their cosmetic properties. For example, leaf and seed extracts [31], supercritical extract of *C. sativa* seed [32], and mixed composition of *C. sativa* plant (leaves, inflorescences, and stem) [33]. However, the callus of *C. sativa* has not yet been examined for its potential as a cosmetic ingredient.

In this study, we highlight the potential of a novel cosmetic composition containing *C. sativa* callus extract (CCE), which serves as an active ingredient with dual anti-inflammatory and antioxidant effects. Enriched with cannabinoid-derived compounds, this extract might offer a promising approach for modulating both oxidative stress and inflammatory responses, making it a valuable candidate for both cosmetic and pharmaceutical applications.

## 2. Materials and Methods

### 2.1. Preparation of Callus Induction Medium

To induce callus formation in *C. sativa*, a Murashige and Skoog (MS) agar medium was prepared (Duchefa Biochemie, Haarlem, The Netherlands). The pH-adjusted (5.6–5.8) medium was then sterilized in an autoclave at 120 °C for 15 min. Thidiazuron (TDZ, MB cell, Republic of Korea) and naphthalene acetic acid (NAA, MB cell, Republic of Korea) were added with final concentrations of 4 mg/L and 2 mg/L, respectively. The medium was then dispensed into Petri dishes (25 mL per dish) and allowed to solidify at room temperature.

### 2.2. Cultivation of Aseptic C. sativa Plants In Vitro

The seeds of *C. sativa* cultivar ‘*Lifter*’ were used in this study. The seeds were sterilized in 70% ethanol for 3 min, followed by disinfection in a 6% sodium hypochlorite solution for 15 min. Residual disinfectants were removed by rinsing the seeds with sterile water. Subsequently, the disinfected seeds were incubated in a 1% hydrogen peroxide solution for 72 h to facilitate germination, and germination was confirmed.

The sterilized and germinated *C. sativa* seeds were sown in culture bottles, with five seeds per bottle, containing a basal MS medium without plant growth regulators. The seedlings were cultured for 14 days under controlled conditions: a light intensity of 142 μmol/m^2^/s (PPFD), a 16 h light/8 h dark photoperiod, and a temperature of 25 °C.

### 2.3. Callus Induction Conditions

To establish optimal callus induction conditions, the *C. sativa* plants cultured in vitro were used. After removing excess moisture, the leaves were excised into 6 mm diameter disks using a biopsy punch. The cultures were incubated for four weeks at 28 °C under a 16 h light/8 h dark photoperiod with a light intensity of 142 μmol/m^2^/s (PPFD).

### 2.4. Preparation of the Callus Extract

**Figure 1:**
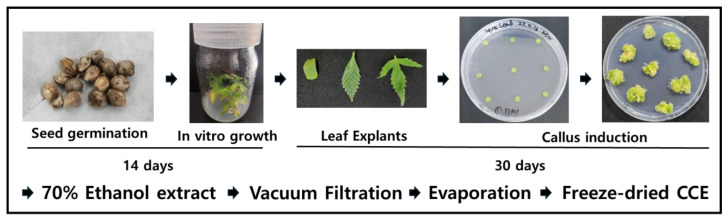
Process of *C. sativa* L. seed germination and leaf tissue-derived callus induction and sample preparation.

To prepare the extract from *C. sativa* leaf-derived callus, the harvested callus was washed and then freeze-dried. The dried callus was pulverized, and 20 g of the powder was extracted in 400 mL of 70% ethanol at room temperature for 72 h. The ethanol was subsequently removed using an evaporator, followed by freeze-drying to obtain the powdered extract. The obtained CCE powder was dissolved in dimethyl sulfoxide (DMSO) at a concentration of 100 μg/mL, filtered, and used for further experiments. The whole process of callus extract preparation is depicted in Figure 1.

### 2.5. DPPH Antioxidant Assay

The antioxidant effects of the CCE were examined by DPPH assay. Specifically, five different sample concentrations were prepared at concentrations of 0, 0.1, 0.5, 1, 5, and 10 mg/mL. 2,2-Diphenyl-1-picrylhydrazyl (DPPH, Sigma, St. Louse, MO, USA) was dissolved in pure ethanol at a concentration of 300 μM.

Next, 100 μL of each prepared sample was mixed with 400 μL of DPPH solution in a reaction tube. The mixture was incubated in a dark condition for 30 min and the absorbance was measured at 517 nm using a microplate reader (Versamax, Molecular Devices, San Jose, CA, USA). As a positive control, Trolox (6-hydroxy-2,5,7,8-tetramethylchroman-2-carboxylic acid, Sigma), a water-soluble derivative of vitamin E was used.

### 2.6. Cell Culture and Treatment

RAW264.7 cells (a mouse macrophage cell line) were purchased from the Korea Cell Line Bank (KCLB, Seoul, Republic of Korea). The cells were cultured in DMEM, supplemented with 10% FBS and 100 U/mL penicillin–streptomycin. Cells were incubated at 37 °C in a humidified 5% CO_2_ atmosphere incubator and maintained at ~70% confluence before being used in the experiments. The cells were co-treated with LPS (1 μg/mL) and the samples for 6 h (for real-time PCR and immunofluorescence microscopy) or 12–24 h (Nitrite determination and Western blot).

### 2.7. Cell Viability Assay

The cell viability of the RAW264.7 cells was determined using an EZ-Cytox assay kit (Daeil Lab, Seoul, Republic of Korea) according to the manufacturer’s instructions. The cells were seeded in 96-well plates in DMEM at a density of 3 × 10^3^ cells per well and then incubated with the medium was replaced with FBS-free DMEM for 24 h. With various concentrations of samples (0–1000 μg/mL) being treated for 24 h, the EZ-Cytox reagent was further treated for 30 min for reaction. The optical densities (ODs) of the reactants were measured at 450 nm using a microplate spectrophotometer (Versamax, Molecular Devices, USA).

### 2.8. Nitrite Determination

The Griess reagent was used to examine the effects of the samples on LPS-induced nitrite levels. RAW264.7 cells (3 × 10^4^ cells/well) were seeded in six-well plates and incubated. At 24 h after seeding, the cells were co-treated with LPS (1 μg/mL) and the samples (0–250 μg/mL) for another 24 h. The supernatants were collected, mixed with the Griess reagent (a mixture of 0.1% N-(1-naphthyl) ethylenediamine (NED) and 1% sulfanilamide dissolved in 5% phosphoric acid) and incubated for 15 min. The ODs were measured at 540 nm using a microplate spectrophotometer. The results obtained from the NaNO_2_ standard curve were used to calculate the nitrite concentrations of reactants.

The fluorescent intensity of intracellular diaminofluorescein (DAF-2) was measured to determine the intracellular NO levels using a DAF-2 fluorescence probe. RAW 264.7 cells (3 × 10^4^ cells/well) were seeded in a 96-well black plate with a clear bottom. Cells were treated with CCE samples and subsequently co-treated with or without LPS (1 μg/mL) followed by the addition of 10 μM of DAF-2 and incubated for 1 h. After incubation, the supernatant was removed from the cells and gently washed. The relative fluorescence intensity was detected at an excitation/emission wavelength of 485/535 nm using a fluorescence microplate reader (SpectraGemini, Molecular Devices, USA). Fluorescence intensities were further examined under an Olympus BX50 fluorescence microscope (Olympus, Tokyo, Japan).

### 2.9. Western Blot

The protein levels of the inflammatory markers and mediators including cyclooxygenase-2 (COX-2), inducible nitric oxide synthase (iNOS), and phosphorylated extracellular signal-regulated kinase (pERK) were determined by Western blot. The RAW 264.7 cells were pre-treated with CCE at concentrations of 50, 100, and 250 μg/mL. Subsequently, the cells were pre-treated with 1 μg/mL LPS for 24 h. Then, the cells were harvested to perform Western blot analysis.

The cells were washed with DPBS (Dulbecco’s phosphate-buffered saline), and the whole proteins were then isolated using ice-cold RIPA buffer containing protease and phosphatase inhibitor cocktail. The protein concentrations in the cell lysates were determined using a commercial BCA kit. The protein lysates (35 μg) were loaded into 7.5% or 10% SDS-PAGE gels, electrophoresed, and transferred to PVDF membranes at 100 V for 60 min using the mini transblot electrophoretic transfer cell device (Bio-Rad, Hercules, CA, USA). The membranes were then blocked with TBS/T (TBS containing 0.1% Tween 20) containing 5% BSA for 2 h, and the blots were incubated with the primary antibody (diluted at 1:1000 in TBS/T containing 3% BSA) overnight at 4 °C with gentle shaking and washed. Subsequently, the membranes were incubated with the secondary antibody (diluted at 1:3000 in TBS/T containing 1% BSA) for 2 h. Chemiluminescent blots were developed using an enhanced chemiluminescence (ECL) buffer (Super Signal West Pico, Thermo Fisher Scientific, Rockford, IL, USA), and blots were detected using a Western blot imaging system (Fusion Solo, Vilber Lourmat, Collegien, France) [34].

### 2.10. Immunofluorescence Staining

The nuclear translocation of NF-κB and NRF-2 was followed by cultivating the cells on chamber slides, using a slight modification of the methodology described elsewhere. The CCE-treated cells (100 and 250 μg/mL) were fixed in 4% formaldehyde for 5 min, permeabilized with 0.1% Triton X-100 for 10 min at room temperature, blocked with 3% BSA for 1 h at room temperature, and labeled with 2 μg/mL of NF-κB primary antibody diluted in 1% BSA overnight at 4 °C. NF-κB in the cytoplasm and nuclei was detected by treating the cells with PBS containing 2 μg/mL of FITC for 1 h at room temperature. Nuclei were stained using a mounting medium containing DAPI (Vector Laboratories, CA, USA) [35]. The fluorescence images were captured under a fluorescence microscope (BX50, Olympus, Japan).

### 2.11. Quantitative Real-Time Polymerase Chain Reaction

qPCR was used to measure the mRNA expression levels of genes associated with pro-inflammatory mediators. Total mRNA was isolated using the Trizol reagent (Invitrogen, Carlsbad, CA, USA) according to the manufacturer’s protocol. The isolated mRNA was subjected to cDNA synthesis using the AccuPower RT Premix kit (Bioneer, Daejeon, Republic of Korea) and oligo (dT)_18_ primers (Invitrogen, Carlsbad, CA, USA). Primer-specific cDNA amplification was performed using a Light Cycler 480 PCR system (Roche, Basel, Switzerland). The PCR reaction mixture contained 10 μL of SYBR green master mix (Roche, Switzerland), 8 μL of ultrapure water, 1 pmol/μL of gene primer, and 1 μL of template cDNA. Amplification was carried out using the following cycle condition: initial denaturation at 95 °C for 10 min, followed by 45 cycles of denaturation at 95 °C for 10 s, annealing at 50–60 °C for 20 s, and extension at 72 °C for 20 s. The following mouse primers were used: TNFα forward, 5′-AAG CCT GTA GCC CAC GTC GTA-3′, reverse, 5′-GGC ACCA CTA GTT GGT TGT CTT TG-3′; IL-1β forward, 5′-CTG AAC TCA ACT GTG AAA TGC CA-3′, reverse, 5′-AAA GGT TTG GAA GCA GCC CT-3′; IL-6 forward, 5′-CCA CTT CAC AAG TCG GAG GCT TA-3′, reverse, 5′-GCA AGT GCA TCA TCG TTG TTC ATA C-3′; β-actin forward, 5′-GCA AGT GCT TCT AGG CGG AC-3′, reverse, 5′-AAG AAA GGG TGT AAA ACG CAG C-3′. β-Actin was used as the internal control, and the results were normalized by dividing the gene threshold cycle values (Ct value) by that of β-actin. All data were acquired using Roche LightCycler 480 software (Roche Applied Science, USA).

### 2.12. ELISA Analysis

Concentrations of inflammatory cytokines including TNFα, IL-1β, and IL-6, in the cell culture supernatants were quantified using Quantikine mouse ELISA kits (R&D Systems, Inc., Minneapolis, MN, USA). Briefly, the cells (5 × 10^5^ cells/well) were seeded in the six-well plates for ELISA and incubated at 37 °C in a humidified atmosphere containing 5% CO_2_. Twenty-four hours after seeding, the cells were co-treated with LPS (1 μg/mL) and CCE samples (0–250 μg/mL) for 24 h. The culture media were then collected, and the TNFα, IL-1β, and IL-6 ELISA were conducted according to the manufacturer’s instructions. The optical densities were measured at 450 nm using a microplate spectrophotometer (Molecular Devices, USA).

### 2.13. HPLC Profile Finerprinting Analysis of CCE

High-performance liquid chromatography (HPLC) was performed using an Agilent 1260 system equipped with UV-DAD, for analyzing the ingredients of CCE.

For chromatographic separation, an Agilent Eclipse XDB-C18 column (4.6 × 250 mm, 5 μm) was used. The mobile phase consisted of two solvents: solvent A (dH_2_O) and solvent B (acetonitrile), applied in an isocratic elution mode over a 30 min run time. The flow rate was maintained at 1 mL/min throughout the analysis. The chromatogram was obtained at a wavelength of 260 nm.

### 2.14. Statistical Analysis

The raw data were analyzed using GraphPad Prism version 5.0 (Graph Pad, La Jolla, CA, USA). One-way ANOVA with Tukey’s multiple comparison test was used to determine the significance of differences. The results are presented as means ± standard deviations (SDs), and *p*-values < 0.05 were considered statistically significant.

## 3. Results

### 3.1. DPPH Radical Scavenging Activity of C. sativa Callus Extract

Antioxidant effect of CCE was examined by DPPH radical scavenging activity. As presented in Table 1, CCE demonstrated significant scavenging activity at 19.39% at a concentration of 2 mg/mL. The summary of the antioxidant activity analysis is presented in Table 1.

### 3.2. Effect of C. sativa Callus Extract on Raw 264.7 Cell Viability

We analyzed the effect of CCE on cell viability. RAW264.7 murine macrophage cells were treated with CCE at concentrations ranging from 25 to 1000 μg/mL. After 24 h of incubation, cell proliferation was assessed using the WST assay.

**Figure 2:**
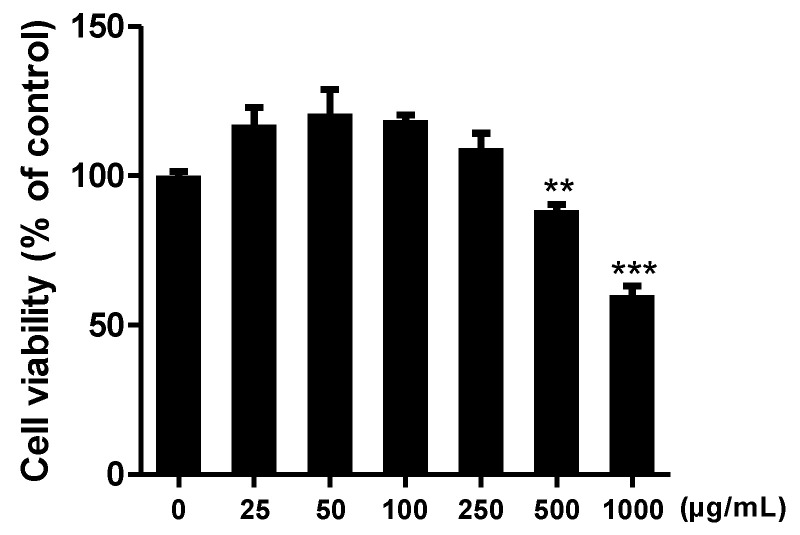
Effect of *C. sativa* L. callus extract (CCE) on Raw 264.7 cell viability at 24 h incubation. The results are presented as the means ± SDs of three independent experiments. ** *p* < 0.01, *** *p* < 0.001, versus untreated controls.

The results indicated that the extract did not significantly affect cell viability at concentrations of 50, 100, 200, and 250 μg/mL. Therefore, these concentrations were selected for further experiments (Figure 2).

### 3.3. Effects of C. sativa Callus Extract on the NO and Pro-Inflammatory Cytokine Production in LPS-Stimulated Macrophages

**Figure 3:**
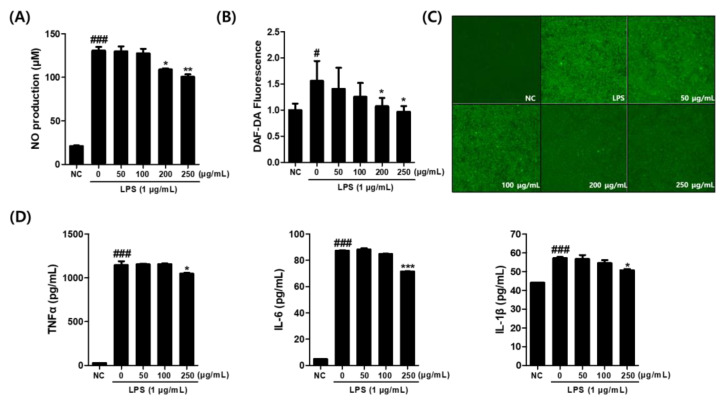
(**A**) Effect of *C. sativa* L. callus extract (CCE) on NO production in LPS-stimulated macrophages. (**B**) Effect of CCE treatment on intracellular nitrite level in LPS-treated macrophages as demonstrated by DAF-DA assay. (**C**) Microscopic analysis of DAF-2 fluorescence images. (**D**) Effect of CCE treatment on production of pro-inflammatory cytokine levels of TNF-α, IL-6, and IL-1β in supernatant of LPS-treated macrophages. The results are presented as the means ± SDs. # *p* < 0.05, ### *p* < 0.001 versus untreated controls, and * *p* < 0.05, ** *p* < 0.01, *** *p* < 0.001, versus LPS-treated RAW264.7 cells.

RAW264.7 cells were treated with the CCE along with 1 μg/mL LPS. The NO released from the cells was measured using the Griess reaction. As presented in Figure 3A, a significant inhibition of nitric oxide production was observed at CCE concentrations of 200 and 250 μg/mL.

When we tested its effects using the DAF-2 fluorescence assay in LPS-stimulated macrophages, CCE significantly reduced intracellular nitrite level, as evidenced by a decrease in relative fluorescence intensity at 200 and 250 μg/mL concentrations compared to the LPS-treated control (Figure 3B). The result was further confirmed by fluorescence intensity measurement obtained from microscopic images (Figure 3C).

Subsequently, the supernatant from the cultured cells was collected, and the levels of inflammatory cytokines TNF-α, IL-6, and IL-1β were analyzed using an ELISA kit. The results confirmed that TNF-α, IL-6, and IL-1β production was induced by LPS treatment. However, when the CCE was treated at 250 μg/mL in the presence of LPS, a significant inhibition of TNF-α production was observed (Figure 3D). Additionally, IL-6 production was markedly suppressed at CCE concentrations of 250 μg/mL. Similarly, IL-1β production was markedly suppressed by CCE treatment at concentrations of 250 μg/mL.

### 3.4. Effects of C. sativa Callus Extract on the Expression of Inflammatory Cytokine Gene Expression Levels in LPS-Stimulated Macrophages

**Figure 4:**
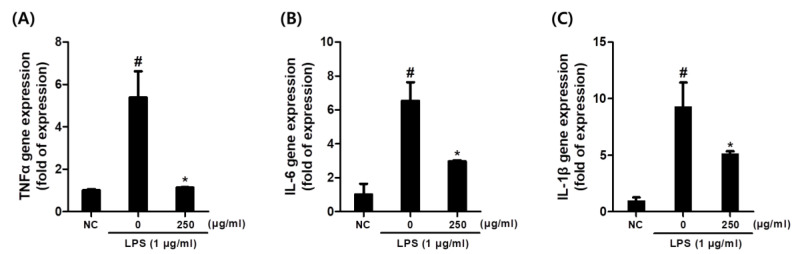
Effect of *C. sativa* L. callus extract CCE treatment on gene expression of pro-inflammatory cytokine in LPS-stimulated macrophages. (**A**) TNF-α. (**B**) IL-6. (**C**) IL-1β. The results are presented as the means ± SDs. # *p* < 0.05 versus untreated controls, and * *p* < 0.05, versus LPS-treated RAW264.7 cells.

Figure 4 demonstrates that gene expression levels of pro-inflammatory cytokines including TNF-α, IL-6, and IL-1β were notably upregulated by LPS activation in macrophages (Figure 4). However, CCE pre-treatment significantly reduced these increases in cytokine transcriptions at a concentration of 250 μg/mL.

### 3.5. Effects of C. sativa Callus Extract on the Inflammatory Mediator Protein Levels in LPS-Stimulated Macrophages

**Figure 5:**
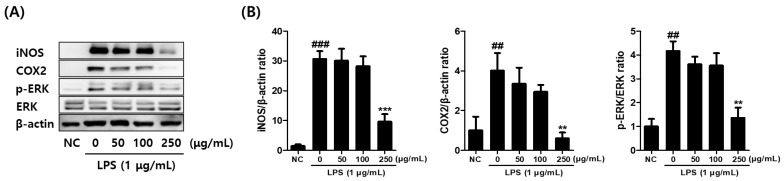
Effect of *C. sativa* L. callus extract (CCE) treatment on inflammatory mediator protein levels demonstrated by Western blot analysis. (**A**) The effect of CCE treatment on the relative iNOS, COX-2, and phosphorylated ERK protein levels in LPS-stimulated macrophages are displayed. The representative band images were presented. (**B**) The band intensities were measured by densitometry and normalized by the intensities of the total forms (ERK) and β-actin. The results are presented as the means ± SDs of three independent experiments. ## *p* < 0.01, ### *p* < 0.001 versus untreated controls, and ** *p* < 0.01, *** *p* < 0.001 versus LPS-treated RAW264.7 cells.

The effects of CCE on the expression of inflammatory mediator proteins, including cyclooxygenase-2 (COX-2), inducible nitric oxide synthase (iNOS), and phosphorylated ERK (pERK/ERK), were analyzed by Western blotting (Figure 5A,B). The results demonstrated that the expression of COX-2, iNOS, and pERK/ERK was significantly induced by LPS stimulation. However, when CCE was co-treated at 250 μg/mL, a significant reduction in all three inflammatory mediator proteins was observed, in a dose-dependent manner (Figure 5B).

### 3.6. Effects of C. sativa Callus Extract on the NF-κB and NRF2 Translocation in LPS-Stimulated Macrophages

**Figure 6:**
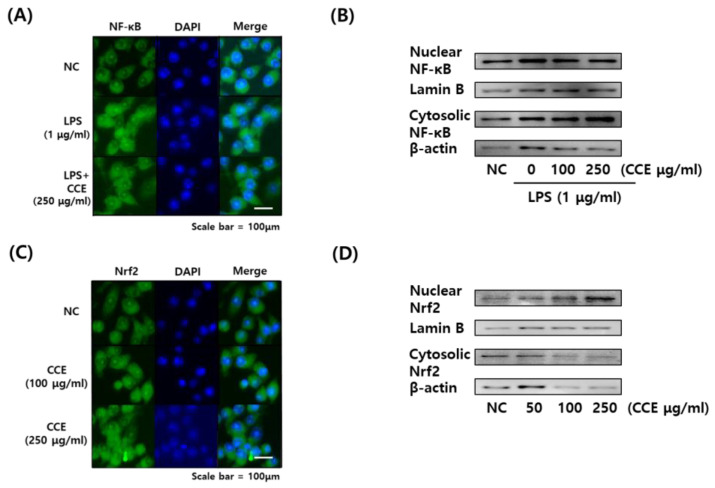
Immunofluorescence microscopy images and Western blot images demonstrating the activation of NF-κB and NRF2 pathways in macrophages by *C. sativa* L. callus extract (CCE) treatment. (**A**) Immunofluorescence microscopy image of NF-κB. (**B**) Western blot image of NF-κB. (**C**) Immunofluorescence microscopy image of NRF2. (**D**) Western blot image of NRF2. (**A**,**C**) NF-κB and NRF2 proteins were labeled with fluorescein isothiocyanate (FITC, green) and the nucleus was stained with DAPI (blue). (**B**,**D**) Representative blot images were presented.

The effects of CCE on NF-κB and NRF2 translocation were investigated by immunofluorescence imaging and Western blot (Figure 6A–D). Immunofluorescence image demonstrated NF-κB translocation to the nucleus by LPS treatment; however, it was inhibited by CCE treatment (Figure 6A). The translocation was further demonstrated by Western blot, which showed decreased phosphorylated NF-κB levels in CCE-treated cells.

In addition, NRF2 fluorescence images demonstrated that the protein was transferred into nucleus by CCE treatment, both in 100 and 250 μg/mL concentrations (Figure 6C). Moreover, its expression level in the nucleus was shown to increase after CCE treatment in a dose-dependent manner (Figure 6D).

### 3.7. HPLC Fingerprinting Analysis of CCE with Cannabinoids

**Figure 7:**
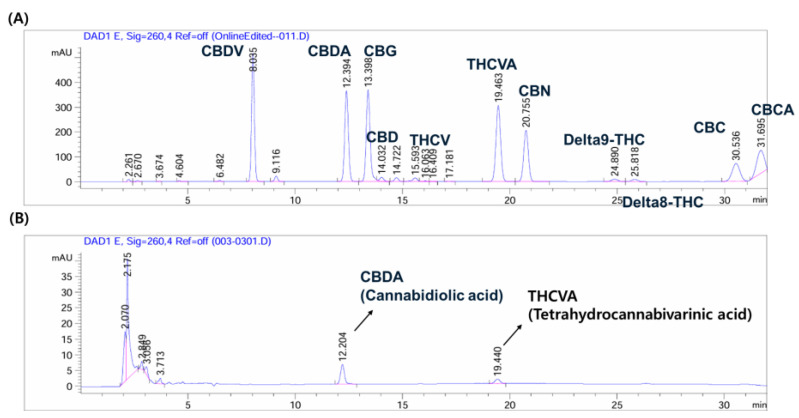
HPLC fingerprinting analysis of *C. sativa* L. callus extract (CCE) compared to the mixed cannabinoid standard. (**A**) Chromatogram of the mixed cannabinoid standard. (**B**) Chromatogram of CCE.

The cannabinoid content of CCE was examined using commercially available mixed cannabinoid standard for analysis. Figure 7A depicts the HPLC profile of various standard compounds and the retention time of cannabidiolic acid (CBDA) was at 12.394 min and tetrahydrocannabivarinic acid (THCVA) at 19.463 min.

Figure 7B depicts the HPLC profile of CCE. As demonstrated, CCE contains a small but significant quantity of CBDA and THCVA.

## 4. Discussion

Plants have long been utilized in the discovery of cosmetic ingredients for centuries [36]. In addition, algae, fungi, and marine organisms have also been explored as alternative sources of bioactive ingredients [36,37]. There remains significant interest in developing strategies to supply new materials for pharmaceutical and cosmetic ingredients. As plants contain a diverse array of bioactive compounds, plant-derived callus can also harbor a wide range of active substances [6]. When integrated with modern plant tissue culture and bioreactor technologies, plant calluses can be mass-produced to meet industrial demands [6]. Therefore, plant calluses, which can be elicited and modifiable for human needs, can serve as a groundbreaking source of new cosmetic materials [38].

In this context, various plant callus extracts were already investigated and evaluated as potential cosmetic ingredients [39,40,41]. Each callus extract has demonstrated effects against oxidative stress and wrinkles [40], skin whitening [39], and skin regeneration [41], depending on its constitution.

Previous studies reported positive effects of *C. sativa* extract with a notable anti-inflammatory and ROS scavenging mechanism [33,42]. Another study found that the *C. sativa* extract inhibits inflammatory mediators involved in skin inflammation and wound healing, which has been attributed to its key component, cannabidiol (CBD) [43]. The active compounds in *C. sativa*, CBD and Δ^9^-tetrahydrocannabinol (THC), are expected to exert their effects by interacting with receptors that modulate the endocannabinoid system [29]. It has been reported that endocannabinoid signaling can impact skin conditions by modulating skin barrier function, inflammation, and unpleasant sensations such as itching and pain [44]. However, it has been reported that the ingredient profile of mature plant extract and callus extract are different, therefore varying their pharmacological efficacy [45,46]. Therefore, further experiments are necessary to determine whether the callus extract of *C. sativa* exhibits similar biological activity to the mature plant extract.

ROS are produced by various harmful stimuli, such as UV, air pollutants, and pathogenic infections in skin lesions [47]. Excessive ROS activation triggers the NF-κB pathway, which regulates the transcription of several matrix metalloproteinases (MMPs), thereby accelerating skin aging [48]. Furthermore, this pathway is strongly associated with chemically induced dermatitis and erythema [49]. On the contrary, the activation of the NRF2/HO-1 signaling pathway can alleviate ROS-related skin conditions by promoting the expression of antioxidant genes [50]. Regulating both pathways is critical for mitigating skin diseases and inflammation.

The results of this study demonstrated that CCE, prepared through an in vitro callus culture method, has significant effects on alleviating inflammatory responses via both intracellular and chemical antioxidant mechanisms. CCE demonstrated strong anti-inflammatory activity by effectively reducing NO production (Figure 3A) and scavenging free radicals (Table 1). In addition, the repetitive impacts of CCE on inhibiting pro-inflammatory cytokines are demonstrated by ELISA (Figure 3D) and mRNA expression (Figure 4A–C). The activities of CCE were assumed to be based on the inhibition of NF-κB and activation of the NRF2 pathway as presented in our data (Figure 6A–D). This suggests that CCE may serve as a potent cosmetic material for addressing skin aging and inflammatory skin conditions, offering dual effects on inflammation condition and oxidative stress.

Previous studies have reported that CBD modulates redox balance, inflammation, and oxidative stress response through Nrf2-NF-κB crosstalk [51]. Therefore, the beneficial effects of CCE might be a consequence of CBD (or acidic forms of cannabinoid) content detected in CCE as demonstrated by HPLC fingerprint analysis (Figure 7B). These properties make CCE a highly promising ingredient for skin-protective cosmetic formulations, given its confirmed CBD functionality and proven broad bioactivity in various diseases.

The cosmetic composition formulated with CCE would exhibit remarkable anti-inflammatory activity, making it a promising ingredient for functional skincare products. In other perspectives, CCE may also serve as a candidate for therapeutic drug development aimed at improving skin conditions or treating inflammatory skin diseases. Moreover, unlike conventional extracts from *C. sativa* leaves or flowers, the use of callus extract is relatively free from regulatory restrictions and utilizes byproducts that would otherwise be discarded, significantly enhancing its industrial applicability.

This study was conducted in vitro, which limits the direct interpretation of the findings to the skin of human subjects. Future clinical trials will be necessary to validate the value of CCE as a cosmetic ingredient with anti-inflammatory and antioxidant properties in a real-world setting. The safety and potential skin irritability of CCE must be assessed in clinical studies to ensure its feasibility as a cosmetic ingredient.

## 5. Conclusions

This study demonstrates that CCE possesses significant anti-inflammatory and antioxidant properties, making it a promising ingredient for skin-protective cosmetic formulations. CCE effectively inhibits NF-κB activation while enhancing NRF2 signaling, contributing to its dual action against inflammation and oxidative stress. The presence of cannabinoid-related compounds further supports its potential therapeutic benefits, as confirmed by HPLC analysis. Future clinical studies are necessary to validate its efficacy and safety, ensuring its applicability in skincare and pharmaceutical industries.

## Figures and Tables

**Table 1:** Evaluation of antioxidant activity of *C. sativa* callus extract (CCE) by DPPH assay.

Sample	Concentration (mg/mL)	Inhibition Rate (%)
*CCE* (*C. sativa* callus extract)	2	19.398 *±* 0.32
	1	15.034 *±* 2.10
	0.2	11.031 *±* 1.86
	0.1	10.008 *±* 1.82
	0.02	7.329 *±* 4.97
Positive Control (Trolox)	2	91.482 *±* 0.15
	1	91.783 *±* 0.15
	0.2	91.693 *±* 0.10
	0.1	91.452 *±* 0.10
	0.02	53.198 *±* 2.76

## Data Availability

All data are contained in this article.
